# USP9X promotes apoptosis in cholangiocarcinoma by modulation expression of KIF1Bβ via deubiquitinating EGLN3

**DOI:** 10.1186/s12929-021-00738-2

**Published:** 2021-06-10

**Authors:** Weiqian Chen, Jingjing Song, Siyu Liu, Bufu Tang, Lin Shen, Jinyu Zhu, Shiji Fang, Fazong Wu, Liyun Zheng, Rongfang Qiu, Chunmiao Chen, Yang Gao, Jianfei Tu, Zhongwei Zhao, Jiansong Ji

**Affiliations:** 1 Key Laboratory of Imaging Diagnosis and Minimally Invasive Intervention Research, The Fifth Affiliated Hospital of Wenzhou Medical University/Affiliated Lishui Hospital of Zhejiang University/Clinical College of The Affiliated Central Hospital of Lishui University, Lishui, 323000 China; 2 Clinical Laboratory, Lishui Central Hospital, Lishui, 323000 China

**Keywords:** Cholangiocarcinoma, Ubiquitination, USP9X, EGLN3, Apoptosis

## Abstract

**Background:**

Cholangiocarcinoma represents the second most common primary liver malignancy. The incidence rate has constantly increased over the last decades. Cholangiocarcinoma silent nature limits early diagnosis and prevents efficient treatment.

**Methods:**

Immunoblotting and immunohistochemistry were used to assess the expression profiling of USP9X and EGLN3 in cholangiocarcinoma patients. ShRNA was used to silence gene expression. Cell apoptosis, cell cycle, CCK8, clone formation, shRNA interference and xenograft mouse model were used to explore biological function of USP9X and EGLN3. The underlying molecular mechanism of USP9X in cholangiocarcinoma was determined by immunoblotting, co-immunoprecipitation and quantitative real time PCR (qPCR).

**Results:**

Here we demonstrated that USP9X is downregulated in cholangiocarcinoma which contributes to tumorigenesis. The expression of USP9X in cholangiocarcinoma inhibited cell proliferation and colony formation in vitro as well as xenograft tumorigenicity in vivo. Clinical data demonstrated that expression levels of USP9X were positively correlated with favorable clinical outcomes. Mechanistic investigations further indicated that USP9X was involved in the deubiquitination of EGLN3, a member of 2-oxoglutarate and iron-dependent dioxygenases. USP9X elicited tumor suppressor role by preventing degradation of EGLN3. Importantly, knockdown of EGLN3 impaired USP9X-mediated suppression of proliferation. USP9X positively regulated the expression level of apoptosis pathway genes de through EGLN3 thus involved in apoptosis of cholangiocarcinoma.

**Conclusion:**

These findings help to understand that USP9X alleviates the malignant potential of cholangiocarcinoma through upregulation of EGLN3. Consequently, we provide novel insight into that USP9X is a potential biomarker or serves as a therapeutic or diagnostic target for cholangiocarcinoma.

**Supplementary Information:**

The online version contains supplementary material available at 10.1186/s12929-021-00738-2.

## Background

Cholangiocarcinoma (CC) is a heterogeneous disease for a complex interaction between host-specific genetic background and multiple risk factors [1]. The incidence rates of cholangiocarcinoma reveal geographical variation in globally, which possess much higher incidence in parts of the Eastern world compared to the West [2]. Cholangiocarcinoma account for 3% of all gastrointestinal tumors, classified anatomically in 3 types: intrahepatic (ICC), perihilar (PCC) and distal (DCC) cholangiocarcinoma [3–5]. Most patients are diagnosed at an advanced, nonsurgical stage and only about 1 in 5 cases are surgically resectable [6, 7]. The outcome of advanced cholangiocarcinoma is poor with an overall survival of maximum 15 months with chemotherapy [8]. Therefore, development of strategies for early diagnosis and effective treatments of cholangiocarcinoma are extremely essential.

Deubiquitylating enzymes (DUBs) play a regulatory role for downstream of ubiquitylation. These post-translational modifiers act as the final arbitrators of a protein substrate's ubiquitylation status, thus regulating its fate [9]. Moreover, DUBs regulate the absolute level of a substrate as well as its locality or activity, beyond being an "all-or-none" phenomenon [9]. Ubiquitin-specific peptidase 9X (USP9X), a member of ubiquitin-specific protease (USP) family, is a highly conserved DUB [10-12]. Accumulating evidence demonstrates that USP9X involves in tumorigenesis and chemoresistance in some types of human cancer, such as breast and lung cancer, melanoma, lymphoma, and glioblastoma [13–16]. However, a tumor suppressor role of USP9X has been documented in pancreatic, colorectal, and renal cancer. The various substrates of USP9X are responsible for its complex role [17-19]. More importantly, the clinical significance and the biological roles of USP9X in cholangiocarcinoma remain unexplored. The purpose of this study was to investigate the function and mechanism of USP9X in cholangiocarcinoma.

The prolyl 4-hydroxylase domain protein 3 (EGLN3), also known as PHD3, is a member of 2-oxoglutarate and iron-dependent dioxygenases [20]. Together with other two closest paralogues, PHD1 and PHD2, these enzymes as cellular oxygen sensors label the hypoxia-inducible factor α (HIF-α) for von Hippel-Lindau protein-mediated proteasomal destruction [20]. Prolyl hydroxylase 3 (EGLN3) is widely accepted as a tumor suppressor [21]. EGLN3 overexpression induces cell apoptosis in a nerve growth factor dependent manner through caspase-3 activation and focal adhesion kinase HIF-1 phosphorylation independently, which participates in the growth and invasion of tumor cells [22, 23]. The kinesin KIF1Bβ was reported acts as downstream from EGLN3 to induce apoptosis [24]. EGLN3 was also documented as a proapoptotic factor in considerable work, including neural crest derivatives, osteosarcoma cells, prostatic carcinoma cells, lung carcinoma cells, and colorectal carcinoma cells [20, 25–30]. Therefore, EGLN3 may play a role as a tumor suppressor in various type of tumors.

## Materials and Methods

### Reagents

The reagents used in this study are listed in Additional file 1: Table S1.

### Immunohistochemical (IHC) staining

Immunohistochemistry (IHC) staining was carried out using EnVision Detection Systems Peroxidase/DAB (DAKO, Shanghai, China) following the manufacturer’s recommendations. Slides containing the sections were stained with commercially available anti-USP9X (ab180191, 1:500, Abcam), anti-EGLN3 (ab238941, 1:500, Abcam) and anti-Ki67 (ab15580, 1:500, Abcam). Two experienced pathologists scored the stained tissues independently. By recording the percentage of positive staining (0 = negative, 1 ≤ 10%, 2 = 10–50%, 3 ≥ 50%) and staining intensity (0 = no, 1 = weak, 2 = moderate, 3 = strong) for each sample, immunoreactivity score (IRS) (0–9) was calculated by multiplying positive staining percentage with staining intensity. Low and high expression were defined according to the median IRS.

### Cell culture and transfection

Human cholangiocarcinoma cell lines RBE and HUCCT were purchased from the Cell Resources Center of Shanghai Institutes for Biological Science, Chinese Academy of Science (Shanghai, China). Cells were cultured in RPMI-1640 (Gibco BRL, Rockville, MD, USA) containing 10% fetal bovine serum (Gibco BRL) in a 5% CO2 incubator at 37◦C. For the gene knockdown assays, cells were infected with lentivirus encoding shRNA respectively.

### Plasmid Construction

Molecular cloning was performed following standard protocols. All construct sequences were confirmed by DNA sequencing. The detailed information concerning expression constructs and the primers used for molecular cloning is provided in Additional file 1: Tables S2 and S3.

### Western blot analysis

Modified RIPA buffer (50 mM Tris–HCl, pH7.4, 1% Nonidet P-40, 0.25% sodium deoxycholate, 150 mM NaCl, and 1 mM EDTA) supplemented with protease inhibitors and phosphatase inhibitors (Bimake, Houston, USA) was utilized to lyse cells. Protein concentrations were detected by BCA protein assay reagent (Yeasen, Shanghai, China). Cellular extracts were resolved through SDS-PAGE, transferred to PVDF membranes (Millipore, Billerica, USA), then incubated with the indicated primary antibodies. Enhanced chemiluminescent substrate kit (Yeasen) was used to analyze corresponding antibody specific signals. Table S4 lists the antibodies used.

### Co-immunoprecipitation (Co-IP)

2 × 10^7^ RBE and HEK293T cells washed by PBS then were harvested and lysed with NP40 lysis buffer (Solarbio, N8031, Beijing, China) containing protease inhibitors cocktail and phosphatase inhibitors (Bimake, Houston, USA). Then the lysates incubated with Flag-, EGLN3-antibody and control IgG after centrifugation respectively in a rotating incubator overnight at 4 °C. Subsequently, the cell lysates were incubated with Protein A/G (Sigma-Aldrich, St. Louis, MO, USA) for another 3 h. Afterwards, the protein A/G Dynabeads were eluted and collected. The eluent was boiled and denatured for immunoblotting.

### Xenograft tumorigenicity assay

Twenty-four 6-week-old BALB/c female nude mice (20 g in average, Shanghai SLAC Laboratory Animal, Shanghai, China) were domesticated in animal facilities for 7 days, and experiments in vivo were carried out. Mice were randomly divided into treatment group (n = 6). Animal research was approved by the Animal Care and Use Committee of Affiliated Lishui Hospital of Zhejiang University. We anesthetized mice (100 mg/kg -10 ketamine, -10, mg/kg, toluthiazide). The flank region of twenty-four BALB/c female nude mice were subject to inject with a total of 3 × 10^6^ cells in 300μL PBS for subcutaneous inoculation. The tumors were measured every 0.5 week after injection then calculated the tumor volume by the formula (length × width2)/2. Throughout the trial, the body weight of each mouse was examined to determine potential toxicity or changes in dose parameters. Clinical symptoms of pain were monitored in all mice during surgery and during daily tumor measurement; no clinical symptoms were observed. The mice were sacrificed 5.5 weeks after inoculation (according to IACUC guidelines, cervical dislocation after carbon dioxide inhalation). Tumor analysis included tumor size and weight.

### Reverse transcription-quantitative PCR

TRIzol agent (Invitrogen, Waltham, MA, USA) was used to extract total RNA then used PrimeScript RT Master Mix to synthesize cDNA (Takara, Shiga, Japan) for Reverse transcription. iQ SYBR Green Master kit (Roche, Shanghai, China) was used to perform qPCR following the manufacturer’s instructions. All data were normalized to the housekeeping gene β-actin, and quantitative measures were obtained by the comparative CT method.

### Colony formation survival and CCK-8 assays

A total of 1 × 10^4^ cells were seeded into 6-well in triplicates for plate colony formation survival assay or 5 × 10^3^ cells were seeded into 96-well in triplicates for CCK-8 assay. Cells were fixed after two weeks by methanol, stained with 0.2% crystal violet solution then photographing for colony formation assays. Colonies consisting of > 50 cells were counted. 10 μL CCK-8 solution (Sigma-Aldrich, St. Louis, MO, USA) was added to each well every week after seeding for CCK-8 assays. The plates were incubated in an incubator for 3 h, and then absorbance at 450 nm was determined.

### CHX assay

100 μg/mL cycloheximide (CHX) was added to cells then harvested at indicated time points for immunoblotting analysis. ImageJ software was used to quantify the densitometry of Western blots.

### Immunofluorescent staining

Four percent methanol-free formaldehyde (Yeasen, #36314ES76) was used to fix cells for 30 min. Cell treated with 0.5% Triton X-100 for 20 min at 4 °C. PBS was used to rinse cells for three times then blocked for 1 h with 5% goat serum and incubated with indicated antibody in 5% goat serum overnight at 4 °C. Cells were rinsed with PBS three times and incubated with the secondary antibodies conjugated with Alexa 488 or Alexa-568 (1:500) at room temperature for 1 h. After washed with PBS for three times, cells were sealed with a DAPI-containing fluoroshield mounting medium (Abcam, #ab104139). Images were visualized with Leica SP5 confocal microscope.

### Cell cycle analysis by flow cytometry

Seventy percent pre-cooled ethanol at 4 °C overnight was used to fix a total of 1 × 10^6^ cells then washed with PBS and subjected to cell-cycle analysis using Cell Cycle and Apoptosis Analysis Kit (Yeasen, #40301ES60) following the manufacturer's instructions. Data were analyzed by FlowJov10 software.

### Statistical analysis

Statistical analyses were performed by SPSS software 24.0 and GraphPad Prism 7.0 (La Jolla, CA, USA). Data were presented as mean ± standard deviation (SD). Student t-test (paired/unpaired) was utilized for values following normal distribution. Chi-Square test was utilized for comparing the differences between categorical variables. The Spearman correlation tests were performed for correlation analysis. The survival curves were gain by Kaplan–Meier method and analyzed by the log-rank test. ROC curve analysis was used to determine the optimal cut-off value of continuous variables. The ANOVA test was performed to analyze the mean values of proliferation rate in different groups. P values of less than 0.05 were considered statistically significant. (*P < 0.05, **P < 0.01, ***P < 0.001, ****P < 0.0001).

## Results

### USP9X expression was downregulated in cholangiocarcinoma patients and correlated with prognosis

**Fig. 1:**
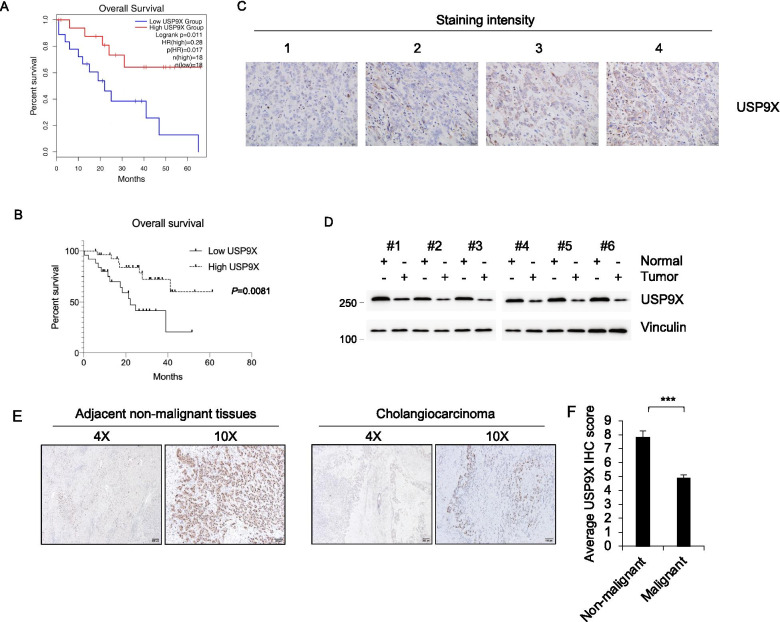
Identification of USP9X Associated with Overall Survival (OS) in Cholangiocarcinoma. **A** Kaplan–Meier curves of OS for 36 cholangiocarcinoma patients from TCGA using GEPIA online website with high or low USP9X expression. **B** Kaplan–Meier curves of OS for 54 cholangiocarcinoma patients with high or low USP9X expression. **C** Representative IHC images of USP9X expression are shown. Scale bars, 50 μm. (D) Six pairs of cholangiocarcinoma tissues and adjacent normal tissues were subjected to immunoblotting analysis with the indicated antibodies. **E, F** 54 cases cholangiocarcinoma tissues and 12 cases adjacent normal tissues were subjected to IHC analysis with the indicated antibodies. Representative IHC images of USP9X expression are shown. Scale bars, 10X 200 μm, 4X 100 μm. ***, p < 0.001

To determine the functional and clinical relevance of genes in cholangiocarcinoma, we first analyzed the genes that affect overall survival (OS) in cholangiocarcinoma from TCGA using GEPIA online website (http://gepia2.cancer-pku.cn). Among these screened genes in the datasets, that patients with high USP9X expression had better OS (Fig. 1A). To validate this finding, we investigated the effect of USP9X expression on postoperative survival in cholangiocarcinoma patients by immunohistochemistry (IHC). Univariate statistical methods were utilized to analyze clinical data from 54 cholangiocarcinoma patients. Patients were allocated to two groups based on the IHC score of USP9X. OS was significantly different between the two groups. The results demonstrated that high expression levels of USP9X are associated with better prognosis of patients with cholangiocarcinoma in OS (Fig. 1B). Representative IHC images are shown in Fig. 1C. These results suggested that the USP9X gene might play a central role in cholangiocarcinoma progression. Furthermore, we evaluated the expression level of USP9X in a cohort of 6 pairs of cholangiocarcinoma and para-tumor (non-cancerous) tissues by immunoblotting. The results suggested that compared with cholangiocarcinoma tissues, USP9X expression level was significantly higher in para-tumor tissues (Fig. 1D). IHC analysis for 54 cases of cholangiocarcinoma tissues and 12 cases of para-tumor tissues also showed that positive staining of the USP9X protein was enriched in para-tumor tissues, but was rarely observed in cholangiocarcinoma tissues (Fig. 1E and F). USP9X expression was found to be correlated with tumor size (Additional file 2: Figure S1A–C). These results suggested that the USP9X gene might play a suppressive role in cholangiocarcinoma progression.

### USP9X suppresses cholangiocarcinoma cells proliferation both in vitro and in vivo

**Fig. 2:**
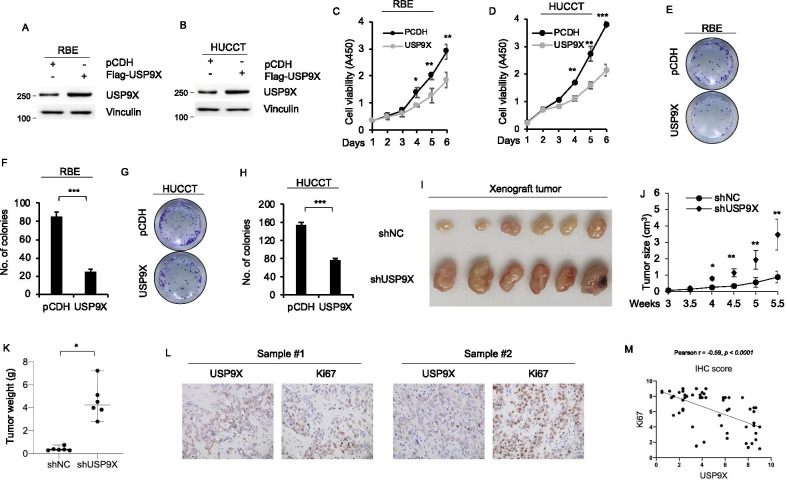
USP9X suppresses cholangiocarcinoma cell proliferation in vitro and tumor growth in vivo. RBE (**A**) and HUCCT (**B**) cells stably expresseing pCDH and Flag- USP9X were analyzed by immunoblotting. RBE (**C**) and HUCCT (**D**) cells stably expressing pCDH and Flag-USP9X was subjected to cell proliferation assays by CCK-8. RBE (**E**) and HUCCT (**F**) cells stably expressing pCDH and Flag-USP9X was subjected to cell proliferation assays by colony growth assays. HUCCT cells stably expressing shNC and shUSP9X #1 were injected into flank region of 6-week-old female BALB/c nude mice (n = 6). After 5.5 weeks of injected, xenograft tumors were harvested. Tumor growth curves (J), photographs of harvested tumors (**I**), and tumor weight (**K**) are shown. **L**, **M** 54 clinical samples were subjected to IHC analysis with the indicated antibodies. Representative images are shown. Scale bars, 20 μm

To investigate the function of USP9X in the progression of cholangiocarcinoma, RBE and HUCCT human cholangiocarcinoma cell lines were overexpressed USP9X (Fig. 2A and B). Cell proliferation assays using CCK-8 kit revealed that overexpression of USP9X inhibited cell proliferation in RBE (Fig. 2C) and HUCCT cells (Fig. 2D). Colony growth assays also indicated that expression of USP9X decreased colony formation of RBE cells (Fig. 2E and F). The same inhibition for colony formation was also observed in USP9X overexpression HUCCT cells (Fig. 2G and H). To evaluate whether the expression level of USP9X could affect cholangiocarcinoma cell growth in vivo, We also established stable USP9X knockdown HUCCT cholangiocarcinoma cells with USP9X shRNA (Additional file 2: Figure S2A). Knockdown of USP9X significantly increase HUCCT cell proliferation in colony formation assay (Additional file 2: Figure S2B–S2C). We subcutaneously injected USP9X knockdown or control USP9X cells into nude mice. The mice were euthanized, compared to control cells, the proliferation rate was significantly increased in USP9X knockdown HUCCT cells (Fig. 2I). The subcutaneous tumors were measured every 0.5 weeks until 5.5 weeks after cell injection (Fig. 2J). The tumor weight was significantly increased in the USP9X-silenced groups than in the control groups (Fig. 2K). Moreover, we also observed that knockdown of USP9X alleviate migration of HUCCT cells (Additional file 2: Figure S2D–E). Furthermore, IHC staining of 54 cases of cholangiocarcinoma tissue suggested that the expression of Ki-67 was markedly downregulated in USP9X high samples (Fig. 2L, M) but upregulated in USP9X low samples, which demonstrated that the expression level of USP9X was negatively correlated with Ki67 expression.

### USP9X interacts with EGLN3 and promotes its expression

**Fig. 3:**
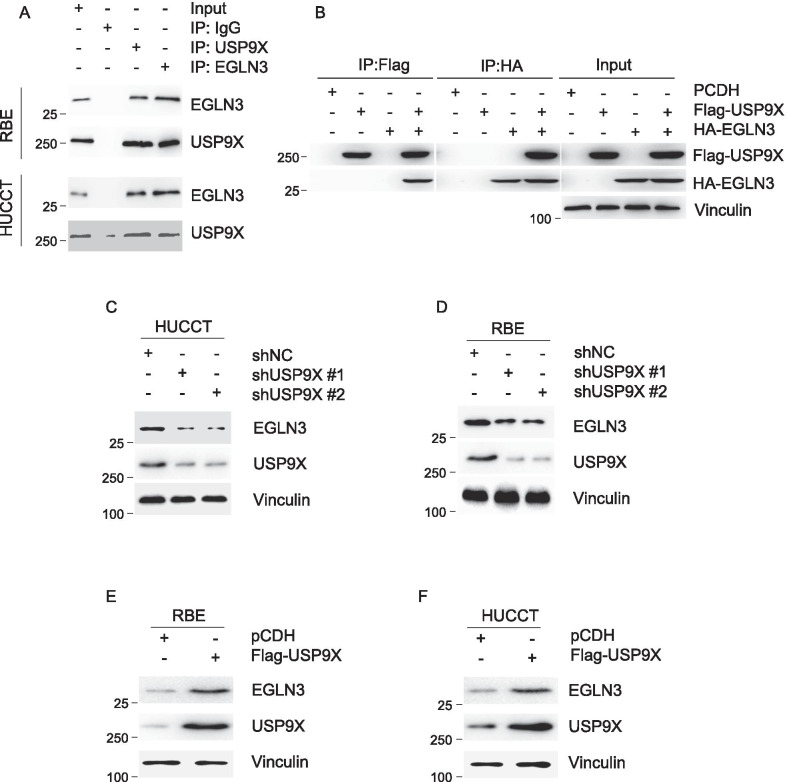
USP9X interacts with EGLN3 and regulates its expressing level. **A** Lysates from RBE and HUCCT cells were immunoprecipitated with control IgG, an anti- USP9X or an anti-EGLN3 antibody, followed by immunoblotting analysis. **B** Lysates from HEK293T cells transiently expressed Flag-USP9X and HA-EGLN3 were immunoprecipitated with anti-Flag or an anti-HA antibody, followed by immunoblotting analysis. RBE (**C**) and HUCCT (**D**) cells stably expressing shNC, shUSP9X #1 and shUSP9X #2 were subjected to analyzed by immunoblotting using indicated antibody. RBE (**E**) and HUCCT (**F**) Cells stably expressing pCDH and Flag-USP9X were analyzed by immunoblotting using indicated antibody

To further characterize the regulatory effect of USP9X on cell proliferation. Integrated bioinformatics platforms (BioGRID and Hitpredict) for investigating the protein interaction network (https://thebiogrid.org) were utilized to predict the substrate of USP9X. We obtained 36 possible interacting with USP9X molecules from these two platforms (Additional file 2: Figure S3A). Five molecules among those that have been clearly reported to be as tumor suppressor. After overexpression of USP9X, we tested the expression levels of these five molecules. The results showed that EGLN3 was increased when USP9X was overexpressed, EGLN3 was selected as a candidate molecule for the next experiment. To achieve this, we next examined whether USP9X interacts with EGLN3. The interaction between USP9X and EGLN3 at the endogenous protein levels was validated in RBE and HUCCT cells by co-immunoprecipitation with an anti- USP9X antibody and anti-EGLN3 (Fig. 3A). Reciprocal IP assays demonstrated that USP9X and EGLN3 formed a ternary complex at the exogenous level in HEK293T cells (Fig. 3B). On the basis of these analyses, we next examined whether EGLN3 expression correlates with USP9X in cholangiocarcinoma cells. We observed that knockdown of USP9X in RBE and HUCCT cells was responsible for the decreased expression level of EGLN3 (Fig. 3C and D). On the contrary, the expression level of EGLN3 was significantly increased after overexpression of USP9X in RBE and HUCCT cells (Fig. 3E and F). Those data indicate that USP9X may play a positive regulatory role on expression of EGLN3.

### USP9X regulates EGLN3 at protein level

**Fig. 4:**
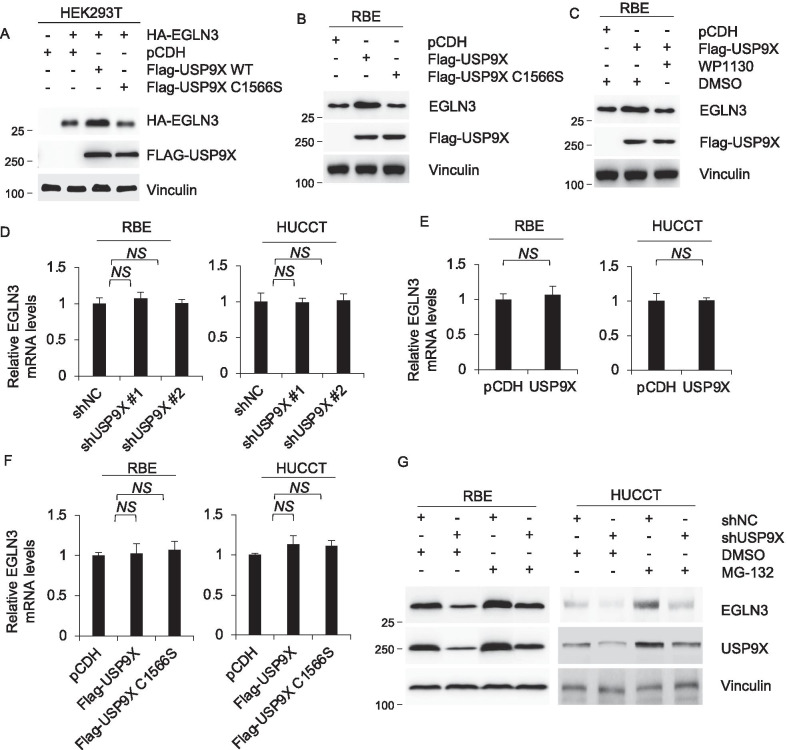
USP9X regulates EGLN3 at level of protein post translational modification. **A** HEK293T cells transiently co-expressing HA-EGLN3 and pCDH, Flag-USP9X WT or FLAG-USP9X C1566S were subjected to analyze by immunoblotting using indicated antibody. **B** RBE cells transiently expressing pCDH, Flag-USP9X WT or FLAG-USP9X C1566S were subjected to analyze by immunoblotting using indicated antibody. **C** RBE cells transiently expressing pCDH or Flag-USP9X WT were added with DMSO or WP1130 then subjected to analyze by immunoblotting using indicated antibody. **D** RBE (left) and HUCCT (right) cells stably expressing shNC, shUSP9X #1 and shUSP9X #2 were analyzed by real-time PCR. **E** RBE (left) and HUCCT (right) cells stably expressing pCDH and Flag-RNF216 were analyzed by real-time PCR. **F** RBE (left) and HUCCT (right) cells added with DMSO or WP1130 were analyzed by real-time PCR. **G** RBE (left) and HUCCT (right) cells stably expressing shNC and shRNF216 #1 were subjected to immunoblotting analysis with the indicated antibodies after 48 h of transfection. Cells were treated with 20 μM MG-132 for 6 h before harvest

To pursue the regulation of EGLN3 by USP9X, we co-overexpress wild-type USP9X and EGLN3 in HEK293T cells. We observed that only wild-type USP9X but not its catalytically inactive mutant (C1566S) upregulated the protein levels of exogenously expressed EGLN3 in HEK293T (Fig. 4A). Compared with wild-type USP9X, catalytically inactive mutant (C1566S) was also deprived of ability for promoting the protein levels of endogenously expressed EGLN3 in RBE cells (Fig. 4B). Similarly, inhibition of USP9X by a partially selective inhibitor WP113046 impaired promotion of EGLN3 by USP9X in RBE cells (Fig. 4C). Those data have been shown that USP9X promotes EGLN3 protein level in a catalytic activity dependent manner. Then, mRNA of EGLN3 were examined by RT-qPCR analysis. Results showed that USP9X depletion by two respective siRNAs did not affect mRNA levels of EGLN3 but significantly reduced its protein levels in HUCCT and RBE cells (Fig. 4D). The increased USP9X also have no influence in mRNA levels of EGLN3 (Fig. 4E). Moreover, the addition of WP113046 made no difference to mRNA levels of EGLN3 (Fig. 4F). USP9X-mediated deregulation of EGLN3 in RBE and HUCCT cells were effectively restored after treatment with 10 μM of proteasome inhibitor MG-132 for 6 h (Fig. 4G). These results indicate the regulation of EGLN3 by USP9X to be posttranscriptional.

### USP9X enhances the stability of EGLN3 and counteracts its ubiquitination

**Fig. 5:**
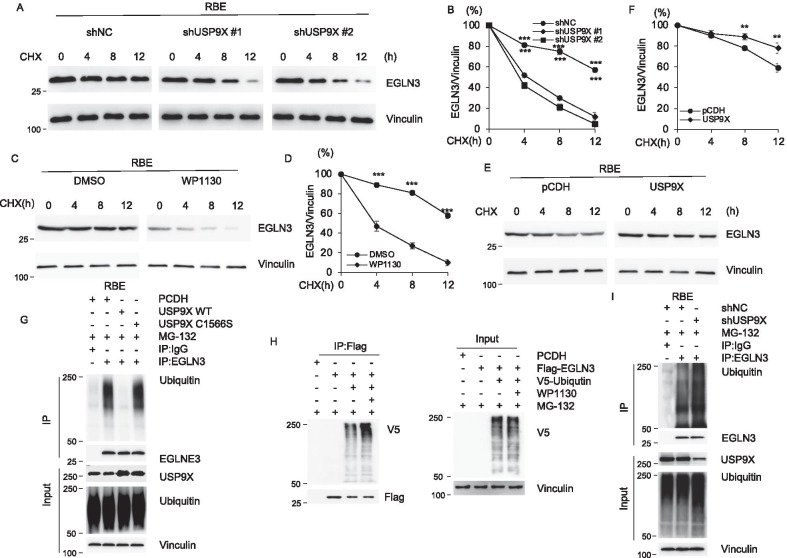
USP9X promotes the deubiquitination of EGLN3. **A**, **B** RBE cells stably expressing shNC, shUSP9X #1 and shUSP9X #2 were subjected to analyze by immunoblotting using indicated antibody. **C**, **D** RBE cells added with DMSO or WP1130 were subjected to analyze by immunoblotting using indicated antibody. **E**, **F** RBE cells stably expressing pCDH and Flag-USP9X were subjected to analyze by immunoblotting using indicated antibody. **G** RBE cells stably expressing shNC and shUSP9X were subjected to IP and immunoblotting analysis with the indicated antibodies after 48 h of transfection. Cells were treated with 20 μM MG-132 for 6 h before harvest. **H** RBE cells were transiently transfected with pCDH and HA-USP9X. IP and immunoblotting analysis were performed with the indicated antibodies after 48 h of transfection. Cells were treated with 20 μM MG-132 for 6 h before harvest. **I** HEK293T cells were transiently transfected with pCDH, Flag- USP9X and V5-Ubiqutin alone or in combination. IP and immunoblotting analysis were performed with the indicated antibodies after 48 h of transfection. Cells were treated with 20 μM MG-132 for 6 h before harvest

To test whether USP9X regulates EGLN3 protein stability, RBE and HUCCT cells stably expressing shNC, shUSP9X #1 and shUSP9X #2 were treated with 100 μg/mL CHX. Samples were collected at the indicated times and then subjected to immunoblotting analysis with the indicated antibodies. As shown in Fig. 5A, B, the half-life of EGLN3 in cells expressing shUSP9X #1 and shUSP9X #2 was significantly shorter than that in cells expressing shNC, indicating that USP9X enhances the stability of EGLN3 protein. Inhibition of USP9X by WP113046 also reduced stability of EGLN3 protein (Fig. 5C, D and Additional file 2: Figure S4A-S4B). On the contrary, overexpression of USP9X enhanced half-life of EGLN3 (Fig. 5E–F and Additional file 2: Figure S4C-S4D). As USP9X is a substrate-specific deubiquitinase, we next examined the effect of USP9X knockdown on EGLN3 ubiquitination. Overexpression of wild-type but not C1566S mutant USP9X in RBE cells decreased the ubiquitination levels of endogenous EGLN3 (Fig. 5G). Moreover, addition of USP9X inhibitor WP113046 showed a significant increase of polyubiquitinated exogenous DIAPH3 protein in HEK293T cells (Fig. 5H) [31]. In agreement with these observations, RBE cells were transfected with shNC and shUSP9X. After 48 h of transfection, cells were treated with 10 μM MG-132 for 6 h and then total cellular lysates were subjected to IP assays with anti-EGLN3 antibody. Immunoblotting analysis showed that USP9X knockdown significantly increased the ubiquitination of EGLN3 protein (Fig. 5I).

### USP9X inhibited growth of cholangiocarcinoma through EGLN3

**Fig. 6:**
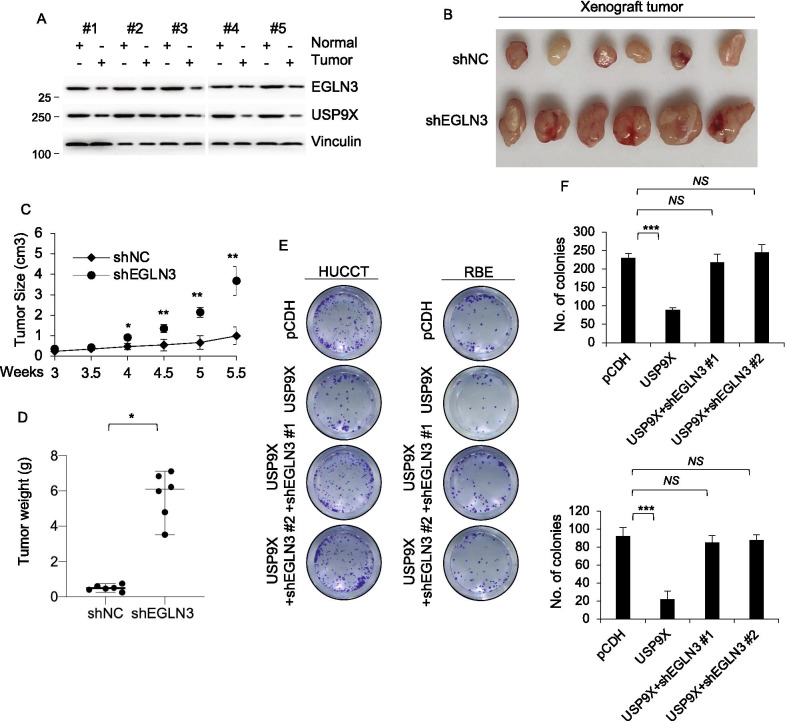
USP9X inhibited growth of cholangiocarcinoma through EGLN3. **A** 5 pairs of cholangiocarcinoma tissues and adjacent normal tissues were subjected to immunoblotting analysis with the indicated antibodies. **B**–**D** HUCCT cells stably expressing shNC and shEGLN3 were injected into flank region of 6-week-old female BALB/c nude mice (n = 6). After 5.5 weeks of injected, xenograft tumors were harvested. Tumor growth curves (**C**), photographs of harvested tumors (**B**), and tumor weight (**D**) are shown. **E**, **F** HUCCT cells stably expressing pCDH, USP9X, shEGLN3 #1, shEGLN3 #2 alone or in combination was subjected to cell proliferation assays by colony growth assays

To further identify the clinical relevance of our findings, we evaluated the correlation of expression levels between USP9X and EGLN3 in 5 pairs of primary cholangiocarcinoma and matched adjacent noncancerous tissues by immunoblotting. As mentioned above, the protein level of USP9X in cholangiocarcinoma tissues was lower than adjacent noncancerous tissues (Fig. 6A). Moreover, the level of EGLN3 in cholangiocarcinoma tissues was lower than adjacent noncancerous tissues (Fig. 6A). These results verified the positive relationship of expression level between USP9X and EGLN3. EGLN3 was documented to inhibit cells proliferation and metastasis in other tumors including prostate cancer, glioma, pancreatic cancer and breast cancer [26, 32, 33]. But, the function of EGLN3 for cholangiocarcinoma remains unknown. Therefore, we examined the function of EGLN3 in cholangiocarcinoma. The results demonstrated that engraft tumors expressing shEGLN3 grew faster than those expressing empty vector (Fig. 6B–D and Additional file 2: Figure S5A). To explore whether tumor growth promoted by USP9X is due to deubiquitinate EGLN3, clone growth assay demonstrated that increased cell proliferation was impaired by knockout of EGLN3 in HUCCT and RBE cell (Additional file 2: Figure S5B-S5F). On the contrary, cell proliferation assays using colony growth assays and CCK8 assay revealed that decreased cell proliferation caused by overexpression of USP9X rescued by knockdown of EGLN3 (Fig. 6E–F and Additional file 2: Figure S5G-S5H).

### USP9X positively regulated the expression level of apoptosis pathway genes

**Fig. 7:**
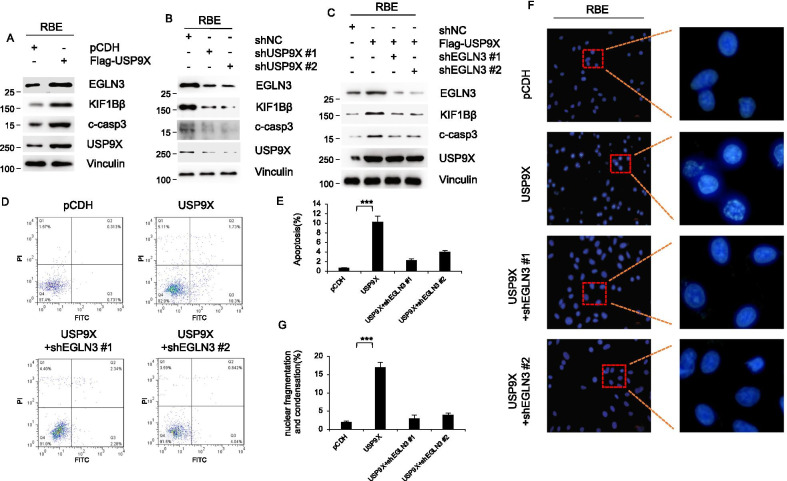
USP9X promotes apoptosis by targeting EGLN3 for proteasomal degradation. **A** RBE cells stably expressing pCDH and Flag-USP9X were subjected to analyze by immunoblotting using indicated antibody. **B** RBE cells stably expressing shNC, shUSP9X #1 and shUSP9X #2 were analyzed by immunoblotting. **C** RBE cells stably expressing pCDH, USP9X, shEGLN3 #1, shEGLN3 #2 alone or in combination were subjected to analyzed by immunoblotting using indicated antibody. **D** RBE cells stably expressing pCDH, USP9X, shEGLN3 #1, shEGLN3 #2 alone or in combination were subjected to analyze by flow-cytometry. **E** RBE cells stably expressing pCDH, USP9X, shEGLN3 #1, shEGLN3 #2 alone or in combination were subjected to analyze by Immunofluorescence laser confocal. Scale bars, 20 μm. ***, p < 0.001

To elaborate the regulatory role of USP9X on the cell proliferation, we further explore the downstream of USP9X and EGLN3. EGLN3 has been reported to promote the expression of KIF1Bβ involved in apoptosis [24]. Frequent deletions of the kinesin-like protein gene 1B (KIF1B) have been reported in neural tumors [34]. Recently, a genome-wide association study demonstrated an association between polymorphisms in the KIF1B gene and the risk of hepatocellular carcinoma (HCC) [24]. Another study documented that downregulation of KIF1B mRNA in hepatocellular carcinoma tissues correlates with poor prognosis [35]. Genetic variations in KIF1B also are reported to contribute to risk of epithelial ovarian cancer (EOC) [36]. These studies suggested that KIF1B may play an important role in the development of other tumors besides nerve tumors. We next validated the regulatory effect of USP9X on components of the apoptosis signaling pathway. Western blot analysis showed that KIF1Bβ and another apoptosis signaling pathway components, as well as apoptosis signaling marker, c-casp3, were increased in USP9X overexpression cells compared to pCDH cells (Fig. 7A). Additionally, shUSP9X treatment resulted in significant decrease of expression level of KIF1Bβ and c-casp3 (Fig. 7B). The increased expression level of KIF1Bβ and c-casp3 promoted by overexpressed USP9X impaired by knockdown of EGLN3 using two respective shRNAs (Fig. 7C). These results suggested that USP9X play a regulatory role on apoptosis signaling pathway through its substrate EGLN3. To further confirm the regulatory role of USP9X on apoptosis signaling, Flow cytometry was utilized to detect apoptosis of RBE cells. Strong positive correlations were observed between USP9X expression and cells apoptosis (Fig. 7D and E). Similarly, the increased apoptosis rate induced by overexpression of USP9X was weaken by knockdown of EGLN3 (Fig. 7D and E). Morphological changes in apoptotic cells provide essential markers for defining and detection of apoptosis as a fundamental mechanism of cell death. Among these changes, the nuclear fragmentation and condensation have been regarded as the important markers. We observed that the rates of nuclear fragmentation and condensation for RBE cells increased significantly after overexpression of USP9X. On this basis, EGLN3 was knockdown using respective shRNAs. As expected, the increased rates of nuclear fragmentation and condensation was eliminated and as much as RBE cells expressing pCDH (Fig. 7F).

**Fig. 8:**
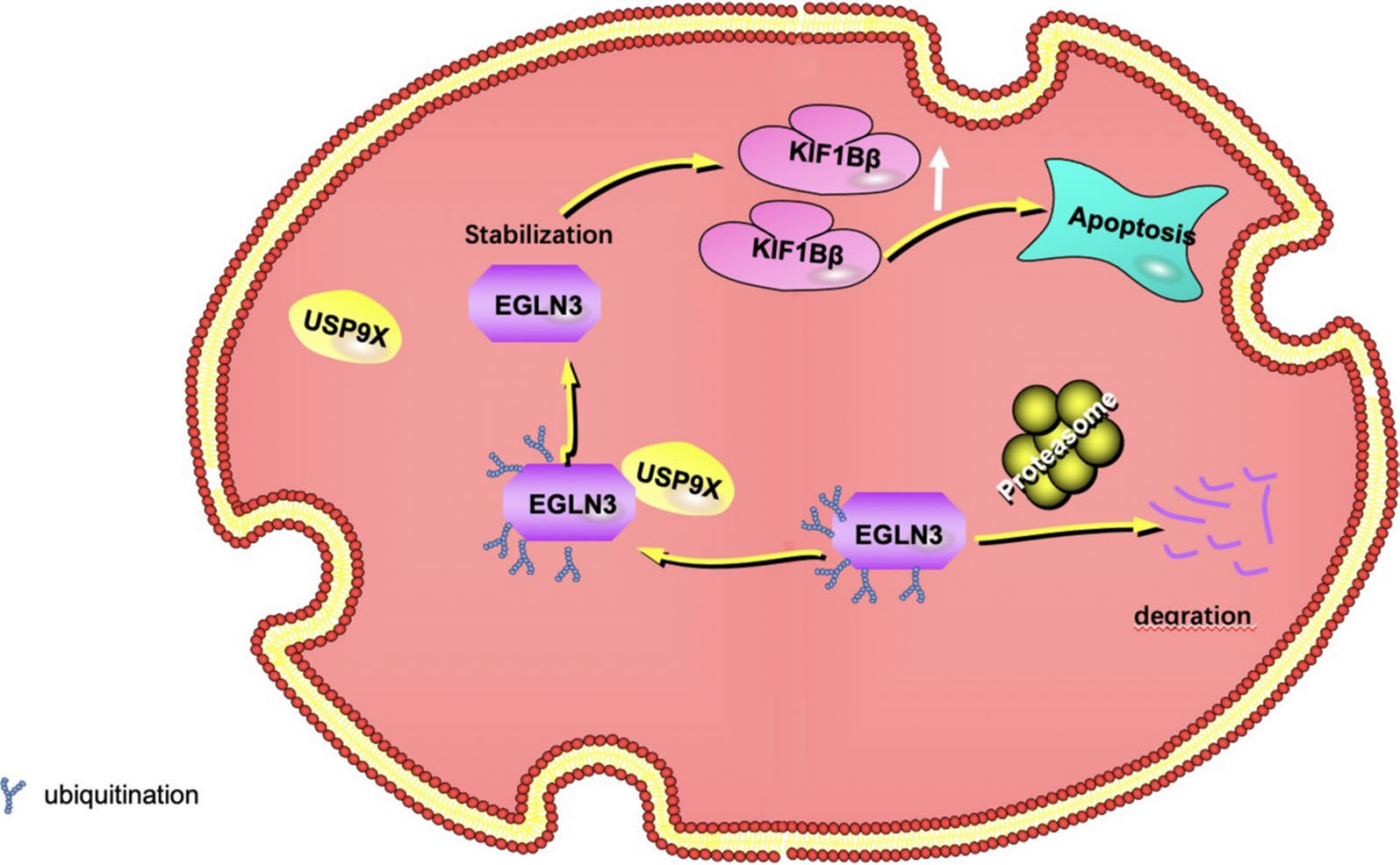
USP9X plays a suppressive role in cholangiocarcinoma progression. USP9X elicited tumor suppressor role by deubiquitinating EGLN3. Knockdown of EGLN3 decimated USP9X-mediated suppression of proliferation. USP9X positively regulated the expression level of apoptosis pathway genes KIF1Bβ through EGLN3 thus involved in apoptosis of cholangiocarcinoma. In conclusion, USP9X plays an inhibitory role in cholangiocarcinoma and serves as a biomarker for the treatment and diagnosis of cholangiocarcinoma

In conclusion, our results unveil USP9X as a potential diagnostic, prognostic and therapeutic tool for cholangiocarcinoma, which provides a novel approach for use in noninvasive screening of cholangiocarcinoma. Findings presented here show that USP9X plays a suppressive role in cholangiocarcinoma progression (Fig. 8). USP9X exerts its tumorigenic suppressive functions through deubquitin-dependent of EGLN3. USP9X functions as a tumor suppressor to participate in apoptosis activation of cholangiocarcinoma. Upregulated DIAPH3 increased the protein level of KIF1Bβ and markers of apoptosis, c-casp3. The suppressive regulation of USP9X in tumor is potentially important. Consequently, evaluating the therapeutic potential of USP9X in cholangiocarcinoma deserve large-scale studies.

## Discussion

Cholangiocarcinoma is the frequent malignancy of the aggressive biliary tract with dismal prognosis [37]. Surgical resection of primary tumor has a better prognosis. However, majority of patients diagnosed at advanced stages thus systemic therapy as the only option [38, 39]. In the past decade, significant progress has been made in comprehending the regulatory mechanism of cholangiocarcinoma tumorigenesis, which pave the way to the precise treatment of this dismal malignancy [40–42].

Diseases of the biliary system, including cholestatic liver disease, cirrhosis and cholelithiasis, increase the risk of cholangiocarcinoma [43–45]. In addition, infectious diseases caused by bacteria, viruses or parasites also increase the risk of cholangiocarcinoma [46, 47]. Other risk factors include toxins, metabolic disorders and some genetic diseases [40, 48]. These risk factors lead to chronic inflammation and/or cholestasis, leading to the activation of common intracellular pathways thus cell proliferation, genetic/epigenetic mutations, and cholangiocarcinoma [48]. Comprehending the molecular pathogenesis of cholangiocarcinoma is essential for the development of new diagnostic biomarkers and targeted treatment of the disease.

A number of mutations have been documented to most likely carry in cholangiocarcinoma [48, 49]. Mutations of dehydrogenase (IDH1, IDH2), fibroblast growth factor receptor (FGFR1, FGFR2, FGFR3), Eph receptor 2 (EPHA2) and BAP1 (genes involved in chromatin remodeling) were reported to present in intrahepatic cholangiocarcinoma, while ARID1B, ELF3, PBRM1, cAMP dependent protein kinase (PRKACA) gene mutations were implicated in the distal and perihilar subtypes subtype [50]. However, more detailed molecular regulatory mechanisms of cholangiocarcinoma are worth profound research. In this study, USP9X modulates the malignant potential of cholangiocarcinoma through regulation of EGLN3. Increased DIAPH3 promoted the protein level of KIF1Bβ and markers of apoptosis, c-casp3. Therefore, USP9X promotes the apoptosis of cholangiocarcinoma cells and alleviates malignant progression of tumor cells. We provided novel insight into the regulatory mechanism and a potential biomarker for cholangiocarcinoma.

Ubiquitination dynamically controlled by ubiqitinating enzymes and deubiquitinating enzymes (DUBs) regulates the stability and turnover of protein [51–53]. The human genome encodes almost 100 deubiquitylating enzymes (DUBs) [54]. Several DUBs, including USP9X, are frequently dysregulated in cancers [19]. USP9X was significantly upregulated in human osteosarcoma cell line SaOS2 expressing prostate-specific antigen [55]. Compared to ERG-negative and benign tumor, USP9X expression is also increased in ERG-positive prostate tumor [56]. Furthermore, ERG was deubiquitylated by USP9X thus stabilized protein levels in prostate cancer cells [56]. However, USP9X suppresses tumorigenesis by stabilizing large tumor suppressor kinase 2 (LATS2) in the Hippo pathway [57]. USP9X promotes FBW7 stability and suppresses colorectal cancer progress [58]. USP9X also is reported as a tumor suppresser in our study. USP9X has tumor suppressor functions via its genetic interaction with Kras. Mutations in KRAS are frequently found in pancreatic ductal adenocarcinoma (PDA), and expression of oncogenic KrasG12D mutation in mouse pancreatic tissue initiates the development of PDA. In these models, genetic inactivation of USP9X (either by insertional mutagenesis or Pdx1-Cre mediated deletion) was found to enhance oncogenic KrasG12D in accelerating tumourogenesis and cancer progression [9]. Consequently, USP9X processes function of both oncogene and tumor suppressor, depending on the type and stage of cancer. Recently, it was reported that loss of USP9X function prevented tamoxifen-induced proliferation arrest in estrogen receptor positive breast cancer cells, which suggested that USP9X is closely involved in the endocrine therapy resistance of breast cancer [31, 59]. Therefore, the regulatory role of USP9X in tumor is diverse.

EGLN3 (also known as PHD3*,* HPH1*,* and SM-20) is a member of the Caenorhabditis elegans gene egl-9 (EGLN) family of prolyl hydroxylases [60]. Lack of mRNA expression of EGLN3 mediates PC3 cells unresponsive to hypoxia [33, 61]. EGLN3 elaborates the growth-suppressive function by suppression of EGFR signaling, which independent to HIF1α and NF-κB in gliomas [62, 63]. EGLN3 arrest cancer cells in G1 phase and mediates apoptosis [32, 64]. These documents support a tumor suppressive role of EGLN3. In our study, Stability and expression of EGLN3 are increased by de-ubiquitination mediated by USP9X. Therefore, USP9X exerts its tumor inhibitory effect through EGLN3. Although the role of USP9X in tumor varies according to tumor type and stage, in our report, we described its inhibitory effect in cholangiocarcinoma, which provides a new therapeutic target and predictor for cholangiocarcinoma.

## Conclusions

In our study, Stability and expression of EGLN3 are increased by de-ubiquitination mediated by USP9X. Therefore, USP9X exerts its tumor inhibitory effect through EGLN3 thus regulated apoptosis pathway by KIF1Bβ in cholangiocarcinoma. A novel pathway through which USP9X / EGLN3 provides promising diagnostic and therapeutic targets against cholangiocarcinoma.

## Supplementary Information

**Additional file 1.** Additional tables.

**Additional file 2.** Additional figures.

## Data Availability

The datasets generated and/or analyzed during the current study are available from the corresponding author on reasonable request.

## Abbreviations

NC: Negative control
sh-NC: NC shRNA
sh-USP9X: ShRNA targeting RNF USP9X
sh-EGLN3: ShRNA targeting EGLN3
RT-qPCR: Reverse transcription quantitative polymerase chain reaction
HEK: Human embryo kidney
Co-IP: Immunoprecipitation
Co-IP: Co-immunoprecipitation
ANOVA: Analysis of variance
FBS: Fetalbovine serum
DMEM: Dulbecco's modified Eagle's medium
FITC: Fluorescein Isothiocyanate
PE: Phycoerythrin
