# Supplementary material for: USP9X promotes apoptosis in cholangiocarcinoma by modulation expression of KIF1Bβ via deubiquitinating EGLN3
Source: J Biomed Sci. 2021 Jun 10;28:44. doi: 10.1186/s12929-021-00738-2 (PMC8191029; doi:10.1186/s12929-021-00738-2)
Supplement: Supplementary file 1 — Additional file 1. Additional tables. [file 12929_2021_738_MOESM1_ESM.docx]

**Table S1. Inhibitors used in this study**

| Chemical inhibitors | Vendors | Cat# | Working concentration |
| --- | --- | --- | --- |
| MG-132 | Selleck | S2619 | 10 μM |
| Cycloheximide | Cell Signaling Technology | 2112 | 100 μg/ml |
| WP1130 | Selleck | S2243 | 5 μmol/L |
| Protease inhibitor cocktail | Bimake | B14002 | 1× |
| Phosphatase inhibitor cocktail | Bimake | B15001 | 1× |

**Table S2. Information for the expression vectors used in this study**

| Plasmids | Sources | Vectors |
| --- | --- | --- |
| Myc-DDK-USP9X | Origene (RC217531) | pCMV6-Entry |
| Flag-USP9X | Subcloned | pCDH-CMV-MCS-EF1-Puro |
| Flag-USP9X C1556S | Subcloned | pCDH-CMV-MCS-EF1-Puro |
| Myc-DDK-EGLN3 | Origene (RC210319) | pCMV6-Entry |
| Flag-EGLN3 | Subcloned | pCDH-CMV-MCS-EF1-Puro |
| HA-EGLN3 | Subcloned | pCDH-CMV-MCS-EF1-Puro |
| Ubiquitin | Hedgehogbio | pCMV |
| V5-Ubiqutin | Subcloned | pCMV6-Entry |

**Table S3. Information for the primer in this study**

| Plasmids | Primers | Sequences |
| --- | --- | --- |
| Flag-USP9X | Forward | GGAATTCATGACAGCCACGACTCGT |
|  | Reverse | TTGATCCTTGGTTTGAGGTGGATCCCG |
| Flag-EGLN3 | Forward | GGAATTC ATGCCCCTGGGACACATC |
|  | Reverse | GTCTTCAGTGAGGGCAGAGGATCCCG |
| V5-Ubiqutin | Forward | CGGATCCATGCAGATCTTCGTGAAA |
|  | Reverse | CGCCTGAGGGGTGGCTGTGAATTCCG |
| Flag-EGLN3 C1556S | Forward |  |
|  | Reverse |  |

**Table S4. Antibody for the primer in this study**

| Antibodies | Vendors | Cat# | Hosts | | Working concentration |  |
| --- | --- | --- | --- | --- | --- | --- |
| USP9X | Abcam | ab180191 | Rabbit | 1: 1500 | | |
| EGLN3 | Abcam | ab184714 | Rabbit | 1: 1500 | |  |
| V5 | Abcam | ab27671 | Mouse | 1: 1500 | |  |
| c-casp3 | Abcam | ab32042 | Rabbit | 1: 1500 | |  |
| CCDC8 | Abcam | ab222969 | Rabbit | 1: 1500 | |  |
| CFTR | Abcam | ab181782 | Mouse | 1: 1500 | |  |
| TERF1 | Abcam | ab10579 | Mouse | 1: 1500 | |  |
| TES | Abcam | ab224375 | Rabbit | 1: 1500 | |  |
| HA | CST | 3724 | Rabbit | 1: 1500 | |  |
| Mouse IgG,Isotype Control | CST | 5415 | Mouse | 1: 1000 | |  |
| Rabbit IgG,Isotype Control | CST | 3900 | Rabbit | 1: 1000 | |  |
| Flag | Sigma | F3165 | Mouse | 1: 1500 (WB)  1: 500 (IF) | |  |
| Vinculin | Sigma | V9131 | Mouse | 1: 3000 (WB) | |  |
| KIF1B | Santa Cruz Biotech- nology | sc-28540 | Rabbit | 1: 1500 | |  |
|  |  |  |  |  | |  |

**Table S5. Information for gRNA**

| gRNA | Sequences |
| --- | --- |
| USP9X gRNA | TGCATTTCCACATACTGACT |

**Figure S1 Identification of USP9X Associated with tumor size in Cholangiocarcinoma**

(A) scatter plots showed the expression levels of RNF216 in 54 cholangiocarcinoma tissues in T1 and T2-T4 stage. (B) IHC staining for 54 samples with cholangiocarcinoma from T1 to T4 stage. (C) Chi-square analysis of the association between USP9X expression and pT stages in cholangiocarcinoma from the 54 samples subjected to IHC staining.

**Figure S2** **USP9X inhibited growth of cholangiocarcinoma**

(A) RBE cells stably expressing shNC and shUSP9X were analyzed by immunoblotting. (B-C) HUCCT cells stably expressing pCDH, shUSP9X #1, shUSP9X #2 alone or in combination was subjected to cell proliferation assays by colony growth assays. (D-E) HUCCT cells stably expressing pCDH, shUSP9X #1, shUSP9X #2 alone or in combination was subjected to cell migration assays.

**Figure S3 USP9X was predicted to interact with EGLN3**

(A) Integrated bioinformatics platforms (BioGRID and Hitpredict) for investigating the protein interaction network (https://thebiogrid.org) were utilized to predict the substrate of USP9X. (B) HUCCT Cells stably expressing pCHD and USP9X were analyzed by immunoblotting.

**Figure S4 USP9X promoted stability of EGLN3**

(A-B) HUCCT cells stably expressing shNC, shUSP9X #1 and shUSP9X #2 were treated with 100 μg/mL of CHX for the indicated times and then analyzed by immunoblotting. Relative expression levels of EGLN3 to Vinculin were shown in below. (C-D) HUCCT cells stably expressing pCDH and USP9X were treated with 100 μg/mL of CHX for the indicated times and then analyzed by immunoblotting. Relative expression levels of EGLN3 to Vinculin were shown in below.

**Figure S5 USP9X inhibited growth of cholangiocarcinoma through EGLN3**

(A) HUCCT cells stably expressing shNC and shEGLN3 were analyzed by immunoblotting. (B-E) RBE and HUCCT cells parental or stably expressing USP9X knockout and EGLN3 alone or in combination was subjected to cell proliferation assays by clone assay (F) RBE and HUCCT cells parental or stably expressing USP9X knockout and EGLN3 alone or in combination was subjected to analyze by immunoblotting
