# Supplementary figures and images for: USP9X promotes apoptosis in cholangiocarcinoma by modulation expression of KIF1Bβ via deubiquitinating EGLN3
Source: J Biomed Sci. 2021 Jun 10;28:44. doi: 10.1186/s12929-021-00738-2 (PMC8191029; doi:10.1186/s12929-021-00738-2)

A

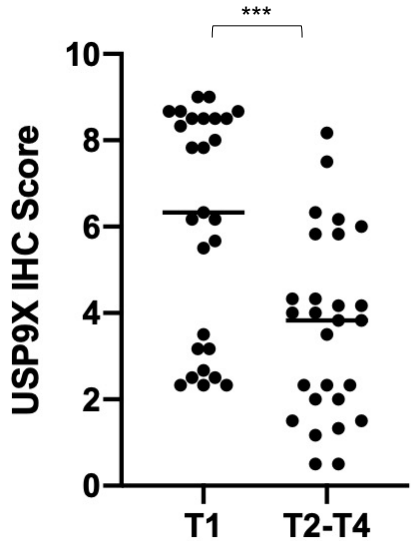

B

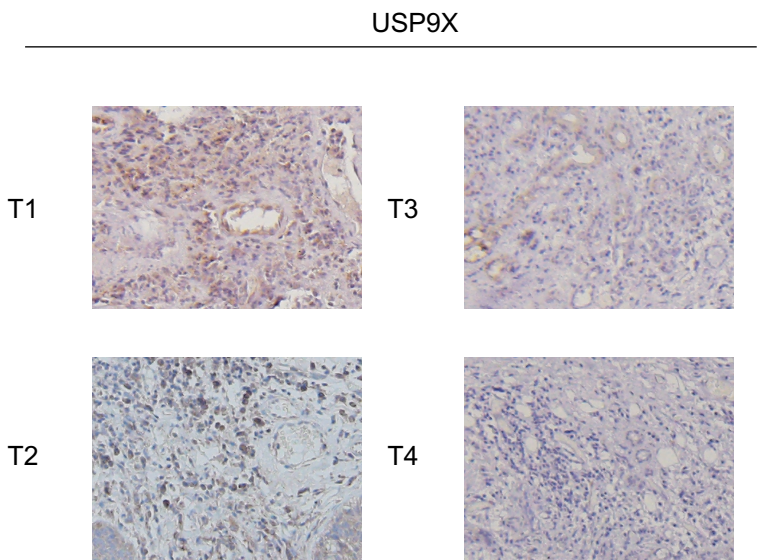

C

| n=54             |      | pT stages     |               |
|------------------|------|---------------|---------------|
| p=0.0027         |      | T1            | T2-T4         |
| USP9X expression | High | 18<br>(72.0%) | 7<br>(28.0%)  |
|                  | Low  | 9<br>(31.0%)  | 20<br>(69.0%) |

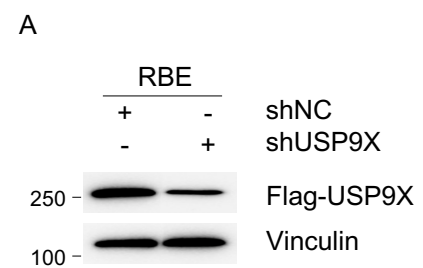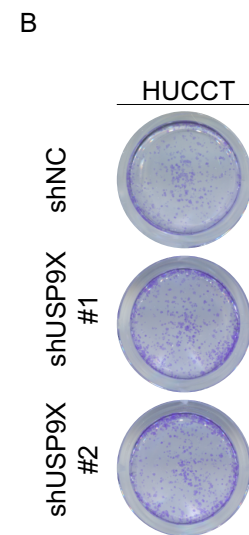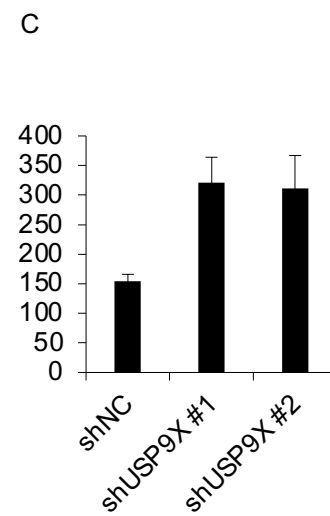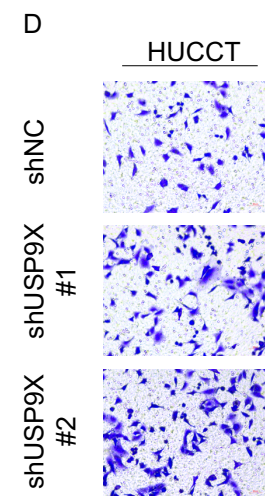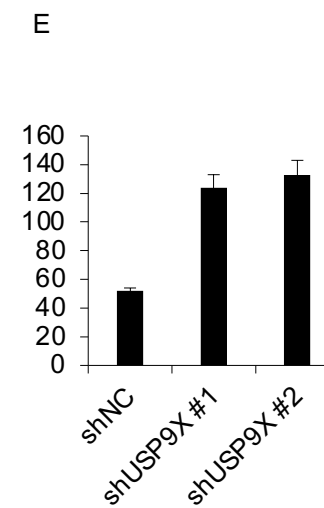

A

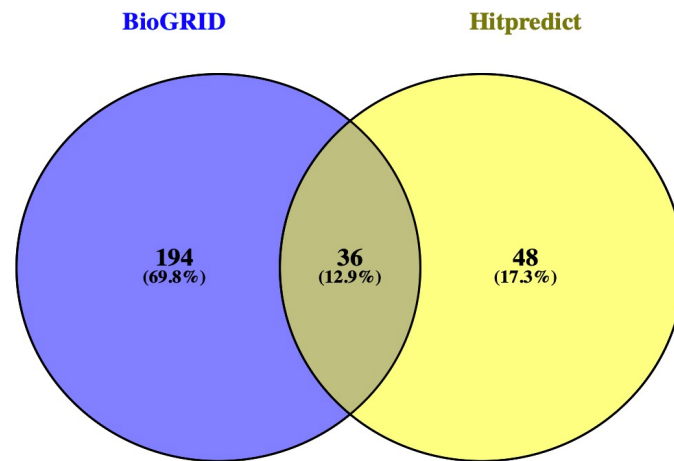

B

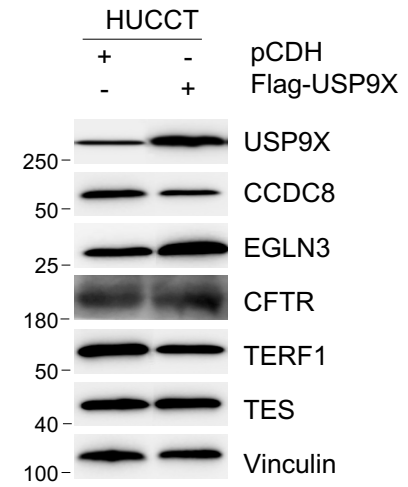

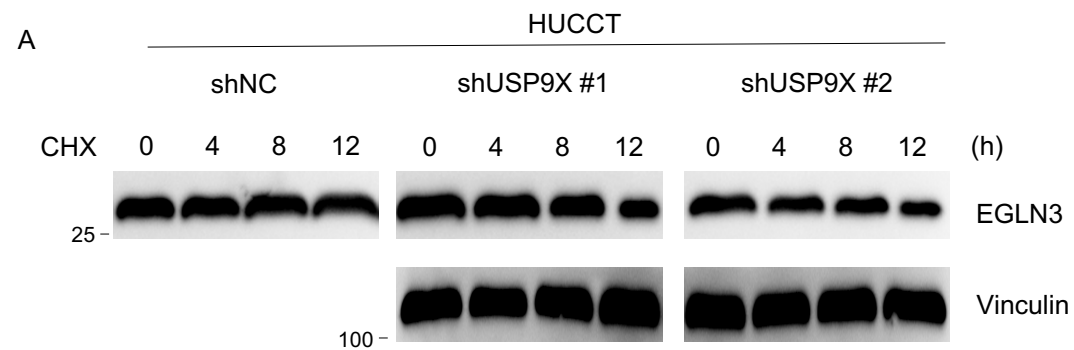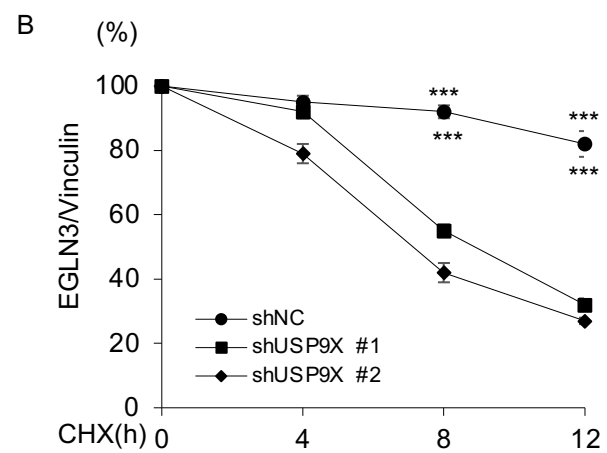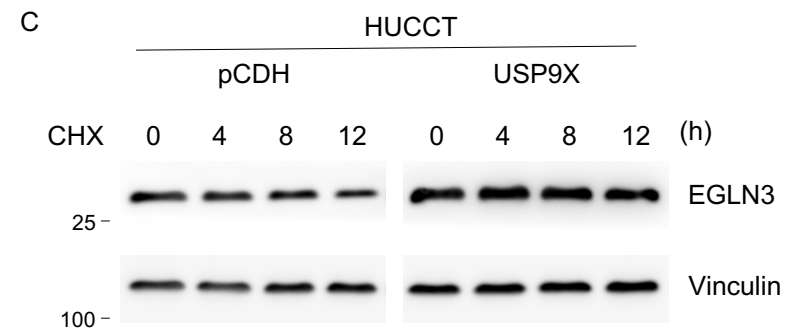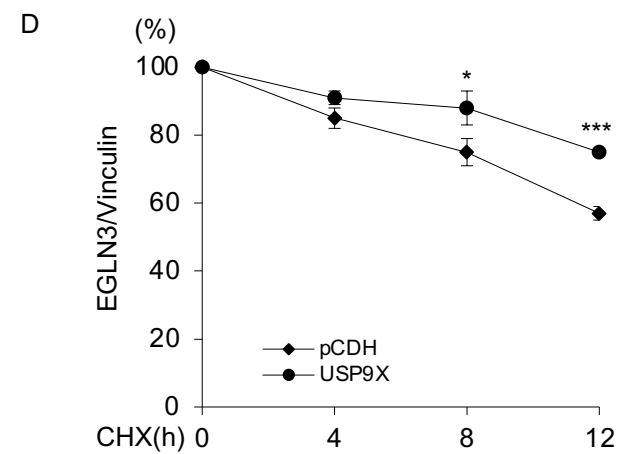

A

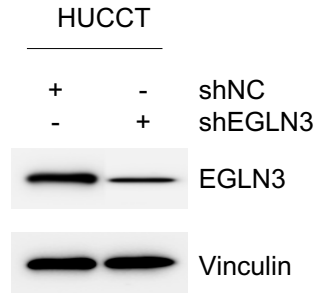

B

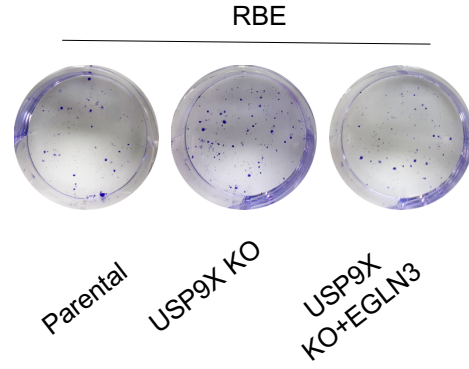

C

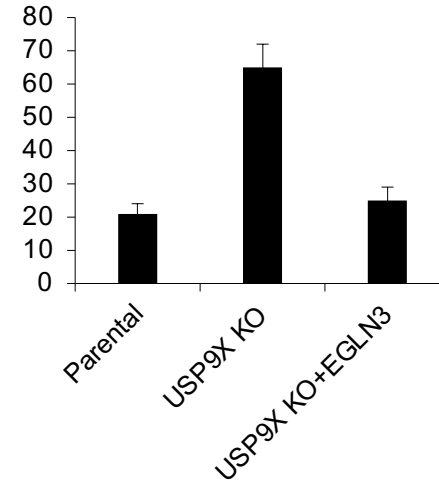

G

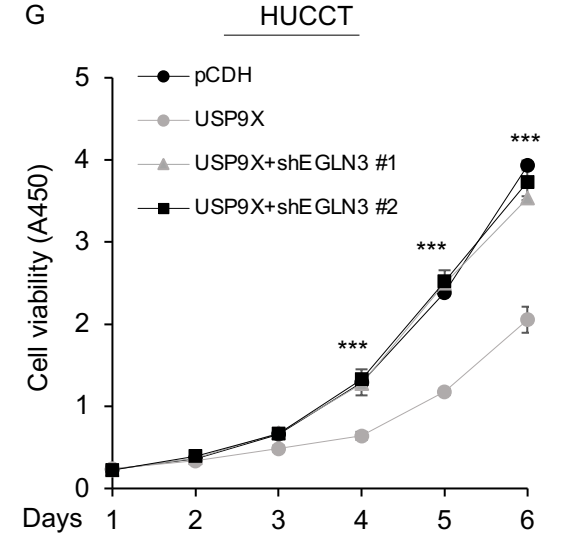

D

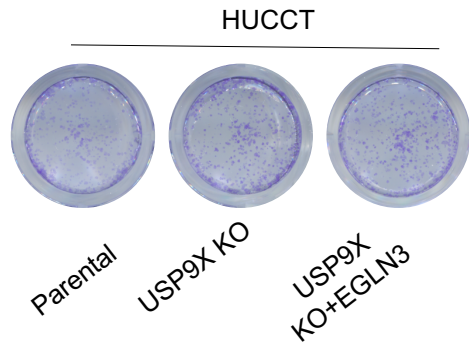

E

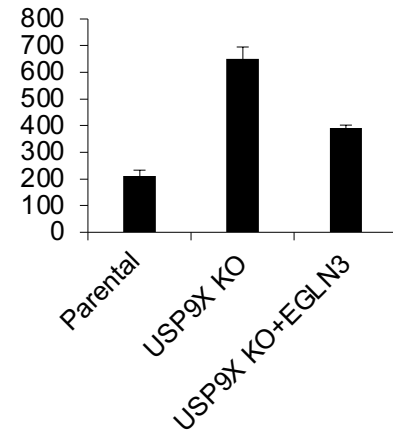

F

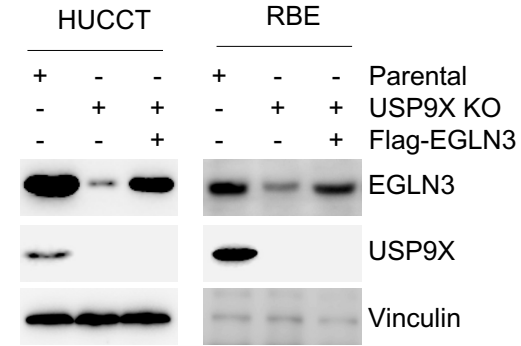

H

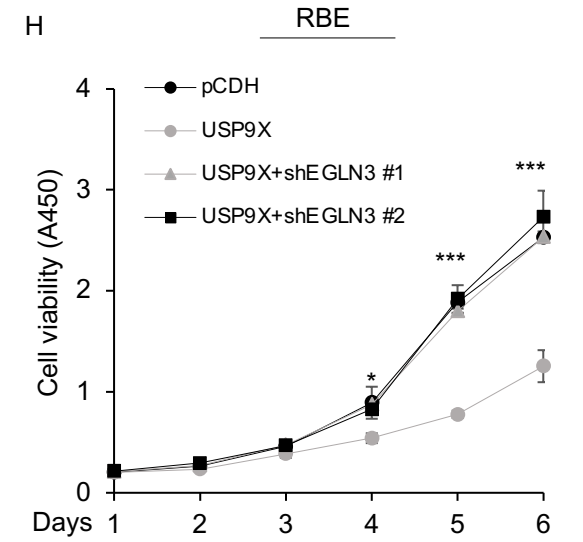

Supplement: Supplementary file 2 — Additional file 2. Additional figures. [file 12929_2021_738_MOESM2_ESM.pdf]
